# Insights From Molecular Dynamics Simulations of a Number of G-Protein Coupled Receptor Targets for the Treatment of Pain and Opioid Use Disorders

**DOI:** 10.3389/fnmol.2019.00207

**Published:** 2019-08-23

**Authors:** João Marcelo Lamim Ribeiro, Marta Filizola

**Affiliations:** Department of Pharmacological Sciences, Icahn School of Medicine at Mount Sinai, New York, NY, United States

**Keywords:** GPCRs, opioid crisis, molecular dynamics, pain, opioid use disorder

## Abstract

Effective treatments for pain management remain elusive due to the dangerous side-effects of current gold-standard opioid analgesics, including the respiratory depression that has led to skyrocketing death rates from opioid overdoses over the past decade. In an attempt to address the horrific opioid crisis worldwide, the National Institute on Drug Abuse has recently proposed boosting research on specific pharmacological mechanisms mediated by a number of G protein-coupled receptors (GPCRs). This research is expected to expedite the discovery of medications for opioid overdose and opioid use disorders, leading toward a safer and more effective treatment of pain. Here, we review mechanistic insights from recent all-atom molecular dynamics simulations of a specific subset of GPCRs for which high-resolution experimental structures are available, including opioid, cannabinoid, orexin, metabotropic glutamate, and dopamine receptor subtypes.

## Introduction

Pain is a vital, albeit unpleasant, physiological response to tissue damage, but it can become a disease if it strikes in the absence of tissue injury, or continues long after appropriate tissue healing (Ringkamp et al., 2018). As a disease, pain poses an enormous socioeconomic burden on the people who suffer from it, as well as a huge financial strain worldwide. There are several different ways to categorize pain (e.g., chronic, nociceptive, neuropathic, etc.) and treatment decisions depend on the specific type of pain (Chang et al., 2015). For severe and chronic pain, the gold-standard painkillers remain opioid drugs, despite their dangerous side effects and abuse liability.

Overprescription of opioid analgesics in the nineties led to drug misuse, and the consequent “opioid epidemic” or “opioid crisis” in the United States, which has most recently expanded to heroin and other illicit synthetic opioids such as fentanyl and its analogs (Volkow et al., 2019). With an average of 130 Americans dying every day (Centers for Disease Control and Prevention [CDC], 2017), new scientific solutions are desperately needed to effectively manage pain while preventing or treating overdose and opioid use disorder (OUD) manifestations. This recognition recently led the leadership of the National Institutes of Health (NIH) and the National Institutes on Drug Abuse (NIDA) to launch initiatives aimed at accelerating the pace of scientific inquiry that is necessary to address the opioid crisis. One of these initiatives enabled the prioritization of specific mechanisms and pharmacological targets whose study is expected to boost the development of novel drugs that have the highest probability of approval by the Food and Drug Administration (FDA) for the treatment of opioid overdose and OUD (Rasmussen et al., 2019). These “most wanted” mechanisms and targets (Rasmussen et al., 2019), which include several G protein-coupled receptors (GPCRs), were established based on published data and internal studies that the NIDA leadership deemed most promising for the development of improved therapeutics for OUDs.

**FIGURE 1:**
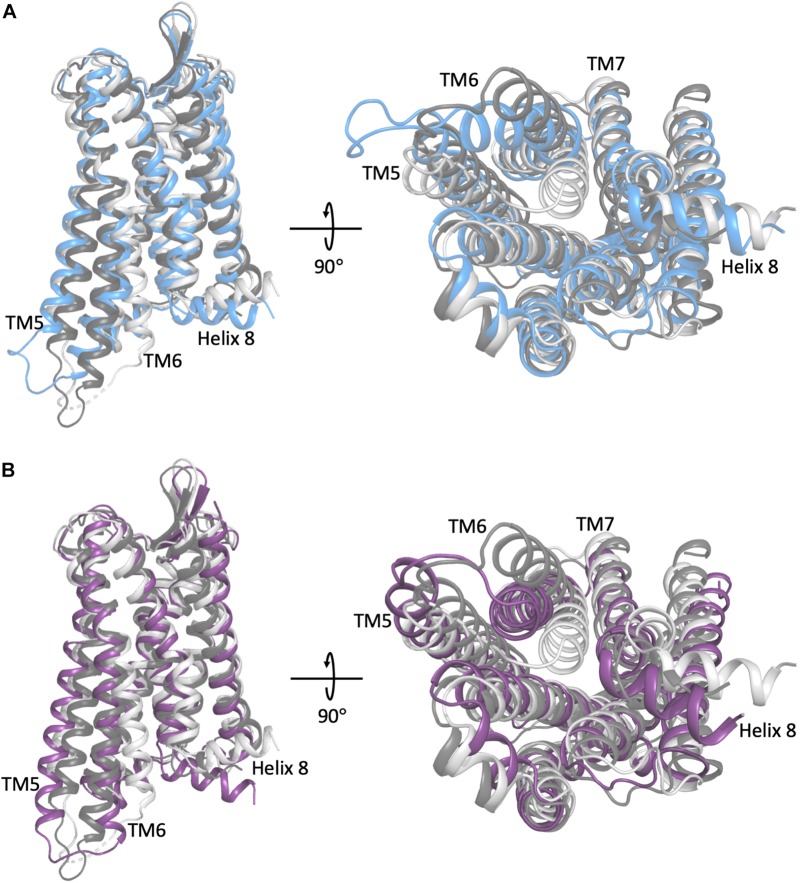
A comparison of the representative conformation of the most probable metastable state within an intermediate region of **(A)** the morphine-bound MOP receptor, where the intermediate state is in light blue, and **(B)** the TRV-130-bound MOP receptor, where the intermediate state is in light purple, relative to the experimentally determined MOP receptor inactive and active states (light and dark gray, respectively). Note that the most dramatic differences between these conformations stem from the extent of outward movement of TM6 away from TM3, which is one of the most notable conformational changes that has been associated with receptor activation. Images on the right correspond to a 90° rotation of the receptor helical bundle, and represent the view from the intracellular domain.

**FIGURE 2:**
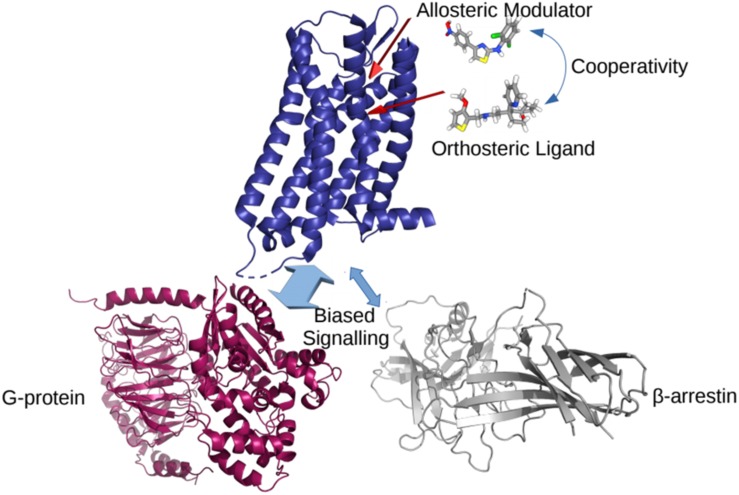
A cartoon representation of the effects of different ligands on a GPCR. The cooperativity between allosteric and orthosteric ligands can shift their affinities for the receptor, and/or bias the GPCR coupling to a particular intracellular partner.

In the classical view of GPCR-mediated downstream cellular signaling, the receptor transitions into active conformational states which are capable of recruiting and ultimately activating intracellular protein transducers such a G-proteins and β-arrestins. These active states are characterized by specific conformational changes at the intracellular end of the receptor, most notably exemplified by a different extent of outward movement of transmembrane helix 6 (TM6) away from TM3 (e.g., see experimentally determined inactive and active structures of a prototypic GPCR compared to intermediate states in Figure 1). Typically, GPCR activation is mediated by the binding of agonist ligands at the so-called orthosteric binding site, which is the same site where endogenous ligands bind. Antagonist and inverse agonist ligands, on the other hand, shift the conformational equilibrium toward inactive conformations of the receptor while partial agonists are expected to stabilize intermediate conformations between inactive and active states of the receptor. For years, drug design at GPCRs has mostly been focused on optimizing ligands for the receptor orthosteric site. However, by binding non-conserved regions of the receptor and directly affecting the binding and/or efficacy of orthosteric ligands, so-called positive and negative allosteric modulators (PAMs and NAMs, respectively) are receiving more and more attention for the development of improved therapeutics targeting GPCRs. Similarly, so-called biased agonists hold a great potential for drug discovery since they would stabilize receptor conformations that selectively recruit an intracellular protein instead of another, thereby triggering specific biological effects. Figure 2 provides a cartoon depiction of the expected effect of the different types of ligands on the receptor.

Indeed, among the NIDA’s ten most wanted medication development priorities in response to the opioid crisis (Rasmussen et al., 2019) are agonists, antagonists, partial agonists, PAMs, and/or NAMs at a number of GPCRs, including orexin-1 or 2, kappa opioid (KOP), GABA_B_, muscarinic M5, nociceptin opioid peptide (NOP), metabotropic glutamate 2/3, ghrelin, dopamine D3, and cannabinoid CB-1 receptors. Additional NIDA-designated priority medications mediated by GPCRs (Rasmussen et al., 2019) included: (i) serotonin 5HT_2__C_ agonists or PAMs, with or without 5HT_2__A_ antagonist/NAM activity, (ii) biased μ-opioid (MOP) receptor agonists or PAMs, and (iii) NOP/MOP bifunctional agonists or PAMs.

**FIGURE 3:**
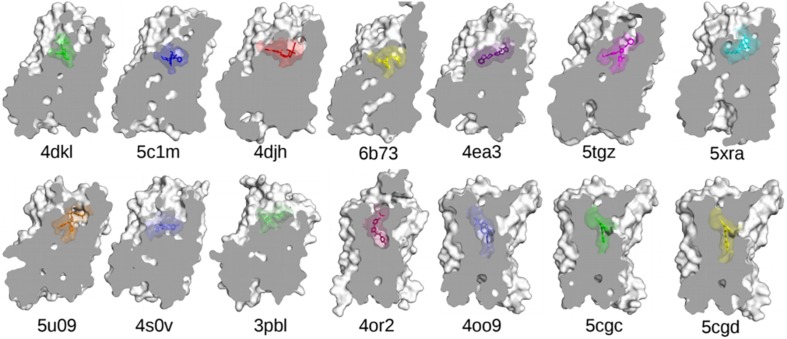
The experimentally determined high-resolution GPCR structures, together with their bound ligands, used in the MD-based studies discussed in this review. Nanobodies and other interacting proteins were removed. PDB 4dkl, The antagonist β-FNA bound to the MOP receptor; PDB 5c1m, The agonist BU72 bound to the MOP receptor; PDB 4djh, The antagonist JDTic bound to the KOP receptor; PDB 6b73, The agonist MP1104 bound to the KOP receptor; PDB 4ea3, The peptide mimetic antagonist compound 24 bound to the NOP receptor; PDB 5tgz, The antagonist AM6538 bound to the CB1 receptor; PDB 5xra, The agonist AM11542 bound to the CB1 receptor; PDB 5u09, The inverse agonist taranabant bound to the CB1 receptor; PDB 4s0v, The antagonist suvorexant bound to the OX2 receptor; PDB 3pbl, The antagonist eticlopride bound to the D3 receptor; PDB 4or2, The negative allosteric modulator FITM bound to the transmembrane domain of mGluR1; PDB 4oo9, The negative allosteric modulator mavoglurant bound to the transmembrane domain of mGluR5; PDB 5cgc, The negative allosteric modulator 3-chloro-4-fluoro-5-[6-(1H-pyrazol-1-yl)pyrimidin-4-yl]benzonitrile bound to the transmembrane domain of mGluR5; PDB 5cgd, The negative allosteric modulator 3-chloro-5-[6-(5-fluoropyridin-2-yl)pyrimidin-4-yl]benzonitrile bound to the transmembrane domain of mGluR5.

One of the main obstacles to the development of new therapeutics for pain management or to treat or prevent opioid overdose or OUDs is the limited understanding of the relevant signal transduction mechanisms at the atomic level notwithstanding the recognized role of a number of GPCRs in the regulation of pain transmission and OUD manifestations, as well as the availability of high-resolution experimental structures for several of these GPCRs. Molecular dynamics (MD) simulations can provide a complementary perspective (Ribeiro and Filizola, 2019) on the molecular determinants underlying GPCR-mediated signaling mechanisms involved in pain transmission, respiratory depression, or clinical manifestations of OUD. Availability of more powerful hardware and software has made the use of MD simulations more affordable and available to a larger number of scientists. It is now straightforward for numerous groups to simulate timescales in the microsecond (μs) regime using high-performance computing resources accessible to a large number of academic institutions. Using either standard or enhanced MD simulations, the latter to access even longer timescales or a more extensive sampling, GPCRs are studied in terms of an ensemble of conformations between fully active and inactive states, with a number of factors, such as binding of ligands, lipids, ions, receptors, or intracellular proteins, shifting the equilibrium toward different states. While we refer the reader elsewhere for an overview of strengths and limitations of MD simulations in their application to GPCRs (e.g., Ribeiro and Filizola, 2019), we review here atomically detailed mechanistic insights from MD simulations of high-resolution experimental structures of a number of GPCR subtypes whose study might lead to faster development of medications for the treatment of pain or OUDs (see Table 1 for a summary of all the MD studies reported herein). These GPCRs include opioid, cannabinoid, orexin, metabotropic glutamate, and dopamine receptor subtypes regulating distinctive pharmacological mechanisms. The position of the co-crystallized ligands in the respective high-resolution experimental structures used as a starting point for the MD simulations referenced herein is shown in Figure 3.

**TABLE 1:** A compilation of the MD-based studies that have been reported in this review article.

**Receptor(s)**	**Ligand(s)**	**Force field**	**Simulation technique**	**Aggregate simulation length**	**References**
MOP	TRV-130, Morphine, Ligand-free	CHARMM36, CGenFF	Unbiased MD	53.25 μs	Schneider et al., 2016
MOP	TRV-130, Morphine	CHARMM36, CGenFF	Adaptive sampling MD	460 μs	Kapoor et al., 2017
MOP	TRV-130, BU72, Naltrexone, β-FNA, Ligand-free	CHARMM36, CGenFF	Unbiased MD	1.5 μs	Cheng et al., 2018
MOP	BMS-986122, (R)-Methadone, Buprenorphine, Ligand-free	AMBER03, Stockholm, GAFF	Unbiased MD	5.2 μs	Bartuzi et al., 2019
MOP, KOP	Morphine, Levallorphan, JDTic, Ligand-free	CHARMM36, CGenFF	Unbiased MD	12.5 μs	Yuan et al., 2015
KOP	5′-GNTI, 6′-GNTI, Ligand-free	CHARMM36, CGenFF	Unbiased MD	1.9 μs	Cheng et al., 2016
KOP	MP1104, JDTic, Ligand-free	AMBER ff14SB, LIPID11, GAFF	Gaussian accelerated MD	12 μs	An et al., 2018
NOP	Cebranopadol, C24, Ligand-free	AMBER ff99SB	Unbiased MD	3 μs	Della Longa and Arcovito, 2019
DOP	BMS-986187, SNC-80	CHARMM36, CGenFF	Metadynamics	3.6 μs	Shang et al., 2016
CB1	THC, THCV, Taranabant, Ligand-free	CHARMM36, CGenFF	Unbiased MD	8 μs	Jung et al., 2018
CB1	CP 55,940, GAT228	CHARMM36, CGenFF	MetaDynamics	–	Saleh et al., 2018
OX2	Suvorexant	AMBER ff98SB, GAFF, Lipid 14	Unbiased MD	400 ns	Bai et al., 2018
OX2	Suvorexant, Nag26, Orexin-A, Ligand-free	AMBER 99sb-ildn, Slipids, GAFF, OPSL-AA	Unbiased MD	36 μs	Karhu et al., 2019
D3R	PF-4363467	CHARMM36, GAAMP	Adaptive sampling MD	680 μs	Ferruz et al., 2018
D2R, D3R	SB269652	CHARMM36, GAAMP	Adaptive sampling MD	76.5 μs	Verma et al., 2018
D3R	LS-3-134, 4 derivatives	AMBER ff14SB, GAFF	Unbiased MD	4.5 μs	Hayatshahi et al., 2018
mGluR1	FITM	CHARMM27, CGenFF	Unbiased MD, Adaptive biasing force	150 ns, 360 ns	Bai and Yao, 2016
mGluR5	Mavoglurant, Dipraglurant, Basimglurant, STX107, MPEP, Fenobam, 51D, 51E	AMBER ff14SB, Lipid14, GAFF	Unbiased MD	800 ns	Fu et al., 2018

## Synopsis of MD Simulation Methods Cited Herein

The goal of this section is not to describe in detail the MD simulation methods and tools cited in this review, but rather provide a lay summary for the general audience. Interested readers are referred to the appropriate reviews, cited below, where the methods are described more thoroughly.

The aim of an MD simulation is to provide the time-evolution of a system by solving the appropriate equation(s) of motion. In these equations, the energy interactions between the particles of the system under study must be described. In principle, atomic-level interactions should be handled using quantum mechanics; however, due to the size of typical biological systems, it is often unfeasible to use the fully quantum description, and classical mechanics is used instead (Oren et al., 2001). The typical approach to modeling these interactions is to describe bonded and non-bonded atomic interactions by simple expressions, with different parameters in these expressions representing different atom types (Oren et al., 2001; Ponder and Case, 2003; Lopes et al., 2015; Nerenberg and Head-Gordon, 2018). The determination of an accurate set of parameters for use in these expressions (the so-called force field) is key to properly describe atomic interactions within biological systems, and it is therefore an intensive area of research. In the following sections, we will mention several different force fields that are currently available to the MD practitioner and have been used in the studies reported here, including those corresponding to the names of Assisted Model Building and Energy Refinement (AMBER) (Maier et al., 2015), Chemistry at Harvard Macromolecular Mechanics (CHARMM) (Best et al., 2012), General Amber force field (GAFF) (Wang et al., 2004), CHARMM General Force Field (CGenFF) (Vanommeslaeghe et al., 2010), etc. In addition, tools such as General Automated Atomic Model Parametrization (GAAMP) (Huang and Roux, 2013) have been developed to automatically generate force field parameters for small molecules not accurately described by the aforementioned force fields.

One of the major obstacles in using MD simulations for investigating biological problems is that the timescale for sampling the event of interest is often larger than the times that can be simulated (Bernardi et al., 2015). While the microsecond (μs) regime is nowadays accessible to a large number of MD practitioners, most biological events fall above that threshold (Valsson et al., 2016; Ribeiro et al., 2019). Enhanced sampling methods are designed to provide a faster exploration of the conformational space of the system under study. In this review, we report on studies carried out using two classes of enhanced sampling MD methods. One of these classes, exemplified by metadynamics and Gaussian accelerated molecular dynamics (GaMD) simulations, uses an artificial biasing force to speed up the rate at which the process of interest is sampled, and so long as this is done in a careful manner, the effect of the “bias” can be “reweighed” to recover “unbiased” information. The other class of enhanced sampling MD simulations is exemplified by adaptive sampling protocols, in which successive batches of simulations are started from regions of conformational space that have been undersampled, thus accelerating the sampling of important, but slow, events (Husic and Pande, 2018; Ribeiro et al., 2019). Throughout the remainder of this review we refer to unbiased MD as simulations in which trajectories are propagated without the help of enhanced sampling techniques, although adaptive sampling techniques are, in principle, not biased (Husic and Pande, 2018).

## Opioid Receptors

Overdose deaths by prescription, illegal, or synthetic opioids have mostly been attributed to the activation of the MOP receptor, a rhodopsin-like (class A) GPCR located, in part, on brainstem neurons that control respiration. In an attempt to develop improved opioid therapeutics with limited respiratory depression and other unwanted side effects (Janecka et al., 2019), attention has recently shifted to G protein-biased agonists of the MOP receptor. These MOP receptor ligands are believed to produce anti-nociceptive action by stabilizing a receptor conformation that preferentially activates G-protein over β-arrestin, the latter shown to be linked to unwanted side effects (Bohn et al., 1999; Raehal et al., 2005).

Recent MD simulations have been leveraged to reveal the molecular details behind G-protein biased agonism at the MOP receptor (Schneider et al., 2016; Kapoor et al., 2017; Cheng et al., 2018). In particular, oliceridine, also known as TRV-130, a G protein-biased MOP receptor ligand that reached phase III clinical trials for management of moderate to severe pain (Viscusi et al., 2019), has been the subject of a number of MD simulations (Schneider et al., 2016; Kapoor et al., 2017; Cheng et al., 2018). Our group, for instance, investigated the binding of TRV-130 from the bulk solvent to the MOP receptor, as well as its preferred mode of interaction at the crystallographically identified orthosteric binding site, using ∼44 μs of unbiased all-atom MD simulations (Schneider et al., 2016). These MD simulations had the MOP receptor placed in a 1-palmitoyl-2-oleoyl-sn-glycero-3-phosphocholine (POPC)/cholesterol lipid membrane environment and used the CHARMM36 force-field to represent the protein and lipid molecules, and CGenFF for the TRV-130 ligand. The results of these simulations suggested that intermediate binding states of TRV-130 at the so-called vestibule region of the MOP receptor directed ligand access to the orthosteric site, and that two energetically indistinguishable conformations could be adopted in the orthosteric binding pocket. Additional microsecond-scale, unbiased simulations of the MOP receptor bound to TRV-130 or the classical orthosteric opioid drug morphine (Schneider et al., 2016) suggested differences in the allosteric coupling between the MOP receptor orthosteric site and the receptor intracellular region induced by the two different ligands. Notably, we found that residues in direct or water-mediated contact with either ligand did not exhibit a main role in the communication between the orthosteric binding site and the intracellular region of the MOP receptor, notwithstanding their contribution to stable ligand binding at the orthosteric pocket. In addition, unlike the morphine-bound receptor, in which the most contributing residues to the allosteric coupling between the orthosteric binding site and the intracellular region of the MOP receptor resided in both transmembrane (TM) helices TM3 and TM6, the TRV-130 complex did not have strong contributors to the co-information in TM6 (Schneider et al., 2016).

To obtain a more thorough investigation of the molecular details of ligand-induced MOP receptor activation, we recently built a Markov state model (MSM) using over 400 μs of MD simulations of the MOP receptor embedded in a POPC/cholesterol membrane mimetic environment with either morphine or TRV-130 bound at the orthosteric binding site (Kapoor et al., 2017). Here, the CHARMM36 and CGenFF force-fields were also used. The MSM revealed that the conformational landscape of the MOP receptor in complex with either ligand contained several kinetic macrostates (i.e., metastable states) in addition to those corresponding to crystal-like active or inactive conformations of the receptor, defining two different intermediate regions of the conformational space for each ligand-MOP complex. These regions contained different conformational states stabilized by morphine or TRV-130, which may or may not get ever resolved experimentally and yet be useful for the rational design of improved opioids with reduced side effects. Shown as an example in Figure 1 are representative conformations of the most probable metastable states within the intermediate regions available to the simulated morphine-bound and TRV130-bound MOP complexes compared to active or inactive crystallographic states of MOP, which are characterized by a different extent of TM6 outward movement. Another important observation of this MSM analysis was the existence of different activation/deactivation pathways induced by the classical or G protein-biased opioid ligand, which confirmed the substantial difference in the receptor dynamics induced by the two different ligands.

In a recent investigation, unbiased MD simulations were used to study the MOP receptor in a ligand-free form, as well as in complex with TRV-130, the agonist BU72, the antagonist naltrexone (NTX), or the antagonist β-FNA. These simulations were each run for 300 ns with the MOP receptor embedded in a POPC membrane environment, the ligands parameters derived from CGenFF, and both the lipid and receptor molecules described by CHARMM36 force field parameters. Notably, simulations of the TRV-130-MOP receptor complex drew attention to two residues in TM6 and TM7, specifically, Y326^7.43^ and W293^6.48^, which had been shown to be important for MOP receptor biased signaling by mutagenesis studies. Superscript residue numbers here and throughout the text refer to the Ballesteros–Weinstein generic numbering scheme (Ballesteros and Weinstein, 1995) wherein the first digit corresponds to the transmembrane helix number and the second digit is a sequence number relative to the most conserved residue in a helix, which is assigned a value of 50. However, corrections to this numbering scheme incorporating structural information have been proposed (Isberg et al., 2015) and will be reported in parenthesis for those residues whose numbering may diverge from Ballesteros-Weinstein’s, such as Y326^7.43^ (renumbered Y326^7.42^ by Isberg et al., 2015).

Similar to MOP receptor agonists, centrally acting KOP receptor agonists can be effective in the treatment of pain, but their dysphoric and hallucinogenic side effects have limited their clinical usefulness (Land et al., 2008), shifting focus to the development of peripherally restricted KOP agonists as analgesics with reduced abuse liability (Hasebe et al., 2004) or KOP antagonists for the treatment of substance use disorders (Carlezon and Krystal, 2016). The structural basis of agonism or antagonism at the MOP and KOP receptors has recently been studied using unbiased all-atom MD simulations (Yuan et al., 2015). A total of four ligand-opioid receptor complexes embedded in a POPC membrane environment were simulated, including the KOP receptor in complex with the JDTic antagonist, the MOP receptor complexed with the agonist morphine, and either the MOP or KOP receptor in complex with levallorphan, a morphinan ligand acting as an antagonist at the MOP receptor and an agonist at the KOP receptor (Yuan et al., 2015). The simulations – each 3 μs in length – made use of CGenFF in their description of the ligands, and the CHARMM36 force field for all remaining molecules. In these simulations, the authors found that the amount of water penetration into the interior of the receptors, which is a known characteristic of GPCR activation, was higher when the receptor was complexed with an agonist as opposed to an antagonist (Yuan et al., 2015). In particular, the levallorphan-MOP and JDTic-KOP complexes formed a σ – π stacking interaction with the Y320^7.43^ (Y320^7.42^ as per Isberg et al., 2015) residue, which tended to block water penetration into the interior. Solvent accessible surface area calculations on subsequent short MD simulations of several other agonists or antagonists in complex with either the KOP or MOP receptors showed these values were higher for receptors in complex with agonists as opposed to antagonists (Yuan et al., 2015).

The conformational changes induced by 6′-Guanidinonaltrindole (6′-GNTI), a G-protein biased agonist that is selective for the KOP receptor, or the antagonist 5′-Guanidinonaltrindole (5′-GNTI) have recently been studied using unbiased all-atom MD simulations (Cheng et al., 2016). In this work, ∼600 ns MD simulations were performed on the ligand-free KOP receptor, as well as the receptor in complex with either 5′-GNTI or 6′-GNTI, with each system embedded in an explicit POPC membrane environment (Cheng et al., 2016). The MD simulations of the KOP receptor bound to the antagonist 5′-GNTI drew attention to the hydrogen bond between S324^7.47^ and V69^1.42^ as the basis for the stabilization of the kink angle on TM7 at about 150°, and possibly deriving antagonistic activity. In contrast, the MD simulation of the G-protein biased agonist 6′-GNTI bound to the KOP receptor showed a different value for this kink angle, and highlighted an interaction of the ligand guanidinium group with the E297^6.58^ residue, together with the steric effect from I294^6.55^, as key contributors to the rotation of TM6, a known hallmark of GPCR activation (Cheng et al., 2016). The possible absence of guanidinium-E297^6.58^ interaction in the MOP or the δ-opioid receptor (DOP) receptor due to this residue replacement by a lysine or tryptophan, respectively, was interpreted as the basis for the lack of 6′-GNTI agonism in those opioid receptor subtypes.

More recently, the KOP receptor conformational changes induced by the agonist MP1104 or the antagonist JDTic were investigated using enhanced sampling MD simulations (An et al., 2018). Specifically, using the generalized AMBER force field for the ligands and the AMBER ff14SB force field for the protein, the GaMD method was used to enhance the sampling of long-time, large-scale conformational rearrangements associated with KOP receptor activation by introducing a biasing harmonic potential on certain dihedral angles. The following systems were all simulated in an explicit POPC membrane environment: the ligand-free KOP receptor in an inactive or active conformation, the latter with or without an intracellular protein, the JDTic-inactive KOP receptor complex, and the MP1104-active KOP receptor complex with or without a stabilizing intracellular partner (An et al., 2018). Taken together, the results of these simulations showed that while the agonist stabilized specific functional domains in an active-like conformation, the antagonist shifted the conformational equilibrium toward an inactive conformation. Notably, the inactive ligand-free state of the KOP receptor was the most stable one, in contrast to the ligand-free active form of the receptor, which readily transitioned to an intermediate state characterized by a reduced TM6 outward movement (An et al., 2018). Finally, the results revealed a hydrophobic interaction between Y246^5.58^ and TM6 in the intermediate metastable state that hindered the transition between the inactive and active conformations of the KOP receptor (An et al., 2018).

The NOP receptor is another opioid target of interest for powerful pain relief with reduced side effects. MD simulations using the AMBER ff99sb force field were recently used to investigate the molecular effect of the novel analgesic cebranopadol (CBP) – which acts as an agonist at both NOP and MOP receptors – on the NOP receptor (Della Longa and Arcovito, 2019). These simulations, run in an explicit POPC membrane environment, used the high-resolution structure of the NOP receptor in complex with the antagonist C24 as a starting point for 1 μs-long simulations of the ligand-free NOP receptor, the C24-NOP receptor complex, and the CBP-NOP receptor complex (Della Longa and Arcovito, 2019). In all cases, the simulations did not sample the large amplitude motions of the transmembrane helices associated with receptor activation even in the presence of the agonist CBP (Della Longa and Arcovito, 2019). The CBP ligand did, however, destabilize the inactive NOP receptor conformation relative to both the ligand-free NOP receptor and the C24-NOP receptor complex such that the NOP receptor bound to CBP could sample a much wider region of the local conformational space (Della Longa and Arcovito, 2019). The authors used these MD simulations to determine some of the earliest microswitches that lead to destabilizing the initial inactive conformation. A histogram of the conformational space of the M134^3.36^ and W276^6.48^ residues located in the orthosteric site revealed that a conformational switch to their active-like positions was accessible to the agonist-receptor complex (Della Longa and Arcovito, 2019). In addition, the time evolution of the conformation of these residues showed that in the agonist-receptor complex these active-like states were “locked” in place for the remainder of the simulation (Della Longa and Arcovito, 2019).

Allosteric modulators of opioid receptors (Remesic et al., 2017) constitute another priority area of research with expected higher probability of success in the development of medications in response to the opioid crisis (Rasmussen et al., 2019). NIDA’s “most wanted” allosteric modulators of opioid receptors include MOP PAMs (Rasmussen et al., 2019). One of the reasons why MOP PAMs are of potential interest is that by increasing the potency and/or efficacy of classical opioid drugs, they are expected to produce the same analgesic response achieved by higher doses of opioid drug while simultaneously presenting fewer on-target overdosing risks. Most importantly, these compounds may not be subject to the compensatory mechanisms deriving from chronic MOP activation (e.g., tolerance, dependence, and increased toxicity) because they preserve the temporal and spatial fidelity of signaling *in vivo* by acting only in the presence of endogenous or other orthosteric ligands (Remesic et al., 2017).

**FIGURE 4:**
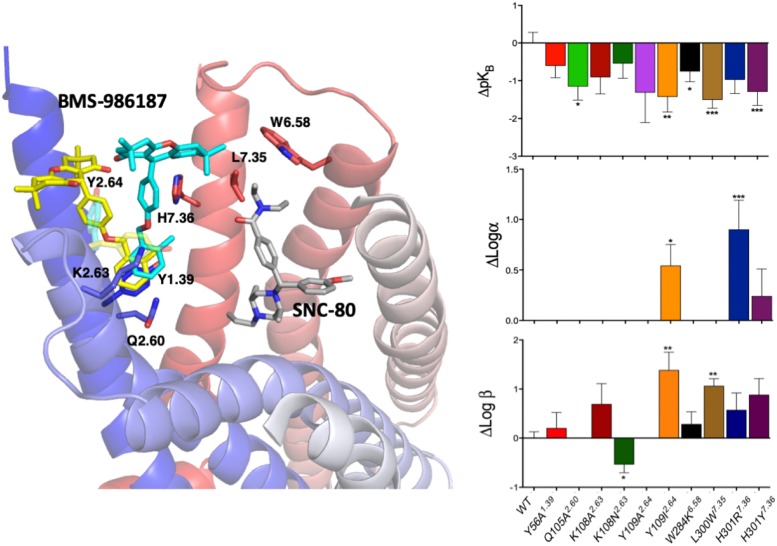
The two energetically most favorable binding modes of the allosteric modulator BMS-986187 – colored yellow and cyan – at the DOP receptor in complex with the orthosteric ligand SNC-80, which is colored in gray. In sticks are the DOP receptor residues in the putative DOP allosteric site used in mutagenesis experiments to help validate the most likely binding mode. The plots to the right show the effect of mutations on the affinity of the allosteric ligand (top right), the induced changes in affinity of the orthosteric ligand (middle plot), and the changes in orthosteric ligand efficacy when the allosteric ligand is bound (bottom right). The experimental data support the predicted BMS-986187 binding mode in cyan color as the most favorable one. Astars indicate the statistical significance levels as given by Dunnet’s test *p* values (^∗^*p* < 0.05, ^∗∗^*p* < 0.01, and ^∗∗∗^*p* < 0.001).

Since experimental high-resolution structures of opioid receptors in complex with allosteric modulators are yet to be published, and automated docking protocols do not yield single binding poses that can be clearly distinguished from the rest, MD simulations can make valuable contributions toward locating allosteric binding sites in opioid receptors, as well as revealing the molecular basis for their binding modes, as we recently demonstrated in an application to the DOP receptor. Specifically, we used metadynamics to simulate the binding of a recently discovered allosteric modulator BMS-986187 of opioid receptors (Burford et al., 2015; Livingston et al., 2018) to the DOP receptor in complex with the orthosteric ligand SNC-80 (Shang et al., 2016). The simulations identified the two most stable binding modes with near-degenerate energies that were discriminated experimentally based on functional studies of normal and mutant receptors (Shang et al., 2016). Figure 4 summarizes the essence of this integrated computational-experimental work, which gave support to the BMS-986187 binding pose in cyan color as the most likely to occur based on the impact of specific mutations (e.g., L/W300^7.35^) on either the intrinsic binding affinity of the PAM or the affinity/efficacy of the orthosteric ligand.

The structural basis for the effect that another allosteric modulator, BMS-986122, has on MOP in complex with either the partial agonist buprenorphine or the agonist methadone, was recently investigated using μs-scale unbiased MD simulations in an explicit membrane mimetic environment (Bartuzi et al., 2019). The results suggested that specific dynamic movements that are characteristic of full receptor activation, such as for instance the bending and rotation of TM7, can be induced by the allosteric modulator even in the presence of a partial agonist at the orthosteric binding site (Bartuzi et al., 2019).

## Cannabinoid Receptors

The cannabinoid receptor 1 (CB1) is another important class A GPCR drug target for the development of new analgesics with reduced side effects. For instance, PAMs of this receptor have been shown to suppress pathological pain without producing tolerance or dependence (Slivicki et al., 2018). These properties, alongside their potentially reduced psychoactive side effects due to their lack of intrinsic activity and inherent ceiling efficacy, make CB1 PAMs potentially better therapeutics for inflammation and chronic pain compared to CB1 orthosteric agonists (Wootten et al., 2013). CB1 antagonists have also been reported to exert analgesia in animal models of inflammatory arthritis and hyperalgesia with reduced side effects (Croci and Zarini, 2007; Ueda et al., 2014). Based on these insights, the CB1 receptor is a target of interest for the development of improved therapeutics to combat the opioid crisis (Rasmussen et al., 2019). Luckily, a number of high-resolution experimental structures for either inactive or active CB1 receptors have been made available (Hua et al., 2016, 2017; Shao et al., 2016; Krishna Kumar et al., 2019) and can be used to study CB1-mediated functional mechanisms at a molecular level for the purpose of guiding rational drug discovery.

A recent unbiased MD simulation-based investigation used these structures in order to gain insight into the features that could explain the different efficacy profiles of partial agonists, antagonists, and inverse agonists bound to the CB1 receptor, and could eventually be used for the design of novel therapeutics targeting the CB1 (Jung et al., 2018). Specifically, analysis of these MD simulations made it possible to discriminate the dynamic tendencies of inactive and active CB1 structures in the presence of ligands with different efficacies while the molecular mechanics Poisson–Boltzmann surface area (MM-PBSA) method was used to assess the contribution of individual ligand-receptor interactions to the binding of the partial agonist Δ^9^-tetrahydrocannabinol (THC), the antagonist Δ^9^-tetrahydrocannabivarin (THCV), and the inverse agonist taranabant from ligand binding free energy decompositions of the CB1-ligand complexes. These MD simulations revealed that binding of the inverse agonist to the active CB1 receptor structure made TM1 less rigid, leading to larger root-mean-square deviations of the possible contacts between TM1 and 2, as well as TM1 and TM7, compared to either the partial agonist-bound or the antagonist-bound active CB1 receptor complex. In addition, the simulations drew attention to large conformational changes involving residues Phe200^3.36^ and Trp356^6.48^ in the orthosteric binding site. In the inactive CB1 receptor, the inverse agonist taranabant – through a direct interaction with Trp356^6.48^ – stabilized the conformation of Trp356^6.48^ and Phe200^3.36^ with respect to one another while the partial agonist THC and antagonist THCV did not. In contrast, in the CB1 active conformation, taranabant induced a different dynamic behavior for the interaction of Trp356^6.48^ and Phe200^3.36^ compared to the partial agonist THC. Furthermore, changes in the binding free energies showed that the partial agonist THC preferred the CB1 active conformation, whereas the simulated inverse agonist taranabant remained more favorably bound to the CB1 inactive conformation via a stable interaction with residue Trp356^6.48^ (Jung et al., 2018) during the afforded simulation timescale.

In another recent investigation, biased MD simulations were used to probe the binding sites and modes of the CP 55,940 agonist and the GAT228 mixed agonist/PAM (so-called Ago-PAM) to the CB1 receptor (Saleh et al., 2018). Ligand binding events to the CB1 receptor were enhanced using the multiple-walker metadynamics biasing protocol (Saleh et al., 2017) and a funnel-shaped restraint applied to the ligand in the bulk, both for the purpose of aiding convergence (Saleh et al., 2018). The simulation of CP 55,940 binding to the ligand-free CB1 receptor – run for 2 μs to achieve convergence – showed a single, deep minimum along the binding potential of mean force (PMF) (Saleh et al., 2018). The location of this deep minimum corresponded to the orthosteric binding site in the high-resolution structure of the CB1 receptor (Saleh et al., 2018). The binding mode of CP 55,940 was found to reproduce all of the interactions observed in the high-resolution structure of the THC agonist AM11542 bound to the CB1 receptor, except for the interaction with F174^2.61^, which was replaced by an interaction with residue F102 in the N-terminal region of the receptor (Saleh et al., 2018). In contrast, the binding simulations of the GAT228 Ago-PAM to the ligand-free CB1 receptor, showed two PMF minima corresponding to binding at different sites of the receptor. These two minima had similar affinities (Saleh et al., 2018), which suggests an equilibrium between binding at the two different receptor sites, thus providing structural context to the experimentally observed partial agonistic effect of GAT228 (Saleh et al., 2018). While the PMF global minimum corresponded to GAT228 bound to the orthosteric site via a cluster of hydrophobic interactions with Val196^3.32^, Leu193^3.29^, and seven additional Phe residues (Saleh et al., 2018), the other PMF minimum defined a putative CB1 receptor allosteric site (Saleh et al., 2018). Notably, simulations of the binding of GAT228 to the CP 55,940-CB1 receptor complex revealed a 3 Å RMSD displacement of the CP 55,940 binding mode induced by GAT228 binding preferentially at an allosteric site defined by residues W279^5.43^, Y275^5.39^, W356^6.48^, and the N-terminus F268 (Saleh et al., 2018), through a hydrogen bond between the indole hydrogen atom of GAT228 and T197^3.33^ (Saleh et al., 2018).

## Orexin Receptors

The orexin (OX) 1 and 2 receptors, expressed throughout the CNS, are neuropeptide receptors that belong to the β-branch of the rhodopsin-like GPCRs (Yin et al., 2016). Although these receptors are known to be important in regulating mammalian sleep patterns (Yin et al., 2015, 2016; Wacker and Roth, 2016), they have recently received attention in the development of therapeutics to address the opioid crisis (Rasmussen et al., 2019). Although high-resolution experimental structures exist for both the OX1 and OX2 receptors bound to antagonists (Yin et al., 2015, 2016; Suno et al., 2018), recent MD-based studies have focused on the OX2 receptor (Bai et al., 2018; Karhu et al., 2019). The earliest of these studies used 200 ns of unbiased MD simulations of the antagonist suvorexant bound to the OX2 wild-type and N324^6.55^A mutant receptors embedded in a POPC lipid environment to understand the dynamic interplay between the horseshoe shape pocket of the receptor revealed by crystallography (Yin et al., 2015) and the boat conformation of the ligand at an atomic level of detail (Bai et al., 2018). In line with the notion of a loss of antagonist binding ability and signaling response in the N324^6.55^A mutant, the results of these simulations showed a distorted horseshoe shape pocket of the N324^6.55^A mutant of the OX2 receptor compared to wild-type receptor, suggesting that an intact horseshoe shape pocket is required for optimal suvorexant binding and antagonistic activity (Bai et al., 2018).

Molecular determinants of OX2 receptor binding and activation were further investigated in a recent MD-based work (Karhu et al., 2019) focused on comparing the receptor dynamic behavior induced by the agonist Nag26 or the antagonist suvorexant, in addition to predicting the mode of binding of the endogenous ligand orexin-A at the OX2 receptor (Karhu et al., 2019). The microsecond-long unbiased MD simulations of Nag26 or suvorexant bound to the OX2 receptor revealed very different dynamic behaviors between the agonist and antagonist, with the agonist exhibiting much increased flexibility and completely different interaction patterns (Karhu et al., 2019). In particular, while suvorexant induced stabilization of the Q134^3.32^–Y354^7.43^ (renumbered Y354^7.42^ according to Isberg et al., 2015) hydrogen bond, Nag26 promoted counterclockwise rotation of the TM5 extracellular end, influencing the interactions among TM4, 5, and 6 (Karhu et al., 2019).

## Dopamine Receptors

The dopamine D3 receptor has also received attention as a drug target for mitigating the opioid crisis (Rasmussen et al., 2019) in large part because of its potential for opioid dependence treatment (Kumar et al., 2016). Recent work on this receptor highlighted the importance of using MD simulations to predict ligand binding at the D3 receptor in agreement with inferences from site-directed mutagenesis (Ferruz et al., 2018). In particular, binding of the antagonist PF-4363467 from the bulk to the dopamine D3 receptor was simulated with ACEMD (Harvey et al., 2009) directed sampling and MSMs generated using High-Throughput Molecular Dynamics (HTMD), using the CHARMM36 force field for the protein and POPC lipid, and ligand force field parameters generated with the GAAMP tool within HTMD. An adaptive sampling protocol was used for these simulations, according to which MSMs were built from successive batches of simulations to identify starting conformations for the next batch, thus affording thorough exploration of the conformational landscape without biasing the potential. A total of over 680 μs of simulation was carried out, resulting in the sampling of two binding paths, which differed in the presence of a second intermediate state in the minor binding path. This intermediate state corresponded to PF-4363467 bound to the D3 receptor at a position between the extracellular vestibule and the orthosteric site. The main structural difference between the predicted PF-4363467-D3 receptor complex and the crystallographically determined eticlopride-D3 receptor complex (Chien et al., 2010) was the formation of an aromatic cryptic pocket between TM5 and TM6 involving residues F338^6.44^, W342^6.48^, L343^6.49^, F345^6.51^, and F346^6.52^ deriving from the displacement of residues F197^5.47^ and F346^6.52^ (Ferruz et al., 2018).

The SB269652 ligand is a bitopic D2 and D3 receptor ligand with negative allosteric modulation activity (Silvano et al., 2010) that has received research attention for the treatment of drug abuse (Verma et al., 2018). By nature of being bitopic, SB269652 binds to both the orthosteric binding site and an allosteric binding site in the receptor. Previous molecular modeling studies suggested that the tetrahydroisoquinoline (THIQ) moiety of SB269652 binds to the orthosteric binding site via an ionic interaction with D^3.32^ whereas the indole-2-carboxamide moiety of SB269652 protruded into a putative allosteric site between TM2 and TM7, forming a hydrogen bond with the E^2.65^ (renumbered E^2.64^ according to Isberg et al., 2015) residue (Guo et al., 2008; Lane et al., 2014). However, mutagenesis and structure activity relationship studies of SB269652 questioned that this hydrogen bond alone could determine the compound allosteric properties (Lane et al., 2014; Mistry et al., 2015; Shonberg et al., 2015), calling for an in depth dynamics study. Thus, adaptive sampling MD simulations using MSMs were recently used to obtain mechanistic insights into the role of the E^2.65^ residue in the binding and allosteric properties of SB269652 at both the D2 and D3 receptors (Verma et al., 2018). Specifically, simulations were carried out for the ligand bound to the wild-type D2 receptor, the E^2.65^A D2 receptor mutant, the wild-type D3 receptor, or the E^2.65^A D3 receptor – all of which were embedded in a POPC lipid environment – for a total of 76.5 μs, with the simulation time of each complex ranging between 15.9 and 21.3 μs (Verma et al., 2018). The THIQ moiety of SB269652 bound at the orthosteric site was shown to be quite stable in both the D2 and D3 receptors, although subtle differences in its binding poses were observed, due in large part to different interactions between the ligand and the extracellular loop 2 (Verma et al., 2018). This is in agreement with previous chimera mutagenesis results that showed the affinities for the D2 and D3 receptors were different in large part due to the extracellular loop 2 (ECL2) (Silvano et al., 2010). In contrast, the indole-2-carboxamide moiety of SB269652 bound at the allosteric site was shown to undergo significant fluctuations, with the MSM analysis revealing two equiprobable metastable states in the wild-type D2 and D3 receptors, but three different metastable states in both the E^2.65^A D2 and D3 receptor mutants (Verma et al., 2018). Furthermore, the results suggested that the E^2.65^ residue mediated the allosteric properties of SB269652 by not only forming a direct hydrogen bond with SB269652, but also by impacting the overall shape and size of the allosteric binding site.

Another recent joint experimental-computational publication also made use of MD simulations to help explain the molecular basis for the binding of bitopic arylamide phenylpiperazine ligands selective for the D3 receptor over D2 (Hayatshahi et al., 2018). Specifically, studies were focused on the prototypic arylamide phenylpiperazine LS-3-134 ligand, which has been found to act as a D3 receptor partial agonist and has also been shown to be 150-fold more selective for the D3 receptor relative to the D2 receptor (Tu et al., 2011). Radioligand binding studies showed that the greatest contribution to the binding energy of the LS-3-134 ligand to the D3 receptor was the phenylpiperazine moiety, but that the arylamide moiety heavily influenced ligand selectivity for the D3 receptor (Hayatshahi et al., 2018). The MD simulations were used to explain the effect that analogs of the piperazine moiety had on the binding affinity. In particular, three different 300 ns unbiased MD simulations were run for LS-3-134 as well as four of its analogs bound to the D3 receptor, revealing that the number of contacts between the protonated nitrogen of piperazine and the D^3.32^ residue tended to decrease as the size of the piperazine increased, with the exception of only one compound. Calculation of their respective binding energies using MM-PBSA showed that the strength of the electrostatic interaction with the D^3.32^ residue generally decreased as the size of the piperazine substituent increased. Umbrella sampling simulations were used to generate a PMF aimed at mimicking the unbinding of the ligand protonated nitrogen from the D^3.32^ residue, and the depth of the bound state in the PMF also agreed with the experimental trend except for one of the analogs (Hayatshahi et al., 2018).

## Metabotropic Glutamate Receptors

Metabotropic glutamate receptors (mGluRs) belong to the glutamate (Class C) subfamily of GPCRs given that their endogenous ligand is the neurotransmitter glutamate. Owing to the demonstrated important role of glutamate in pain sensation and transmission, these receptors have been suggested to be promising potential targets for novel pain relieving medications (Pereira and Goudet, 2018). There are eight different subtypes of mGluRs, which are divided into group I (mGluR1 and 5), group II (mGluR2 and 3), and group III (mGluR4, 6, 7, and 8) receptors. While group I mGluRs signal via G_α__q_, groups II and III signal via G_α__i_. Several articles suggest opposing effects of the group I vs. group II and III receptors in reference to antinociception, with group I antagonists or group II/III agonists in the spotlight from a drug discovery perspective. In particular, mGluR2/3 agonists or PAMs have been listed among NIDA’s “most wanted” medications in response to the opioid epidemics (Rasmussen et al., 2019).

Like other class C GPCRs, mGluRs are structurally different from rhodopsin-like (class A) GPCRs in that they have a large extracellular domain, also known as Venus Flytrap Domain (VFD), in addition to the 7 transmembrane helical domain (7TMD). Unlike class A GPCRs, the endogenous ligand binding site of mGluRs is located in the extracellular VFD, whereas the transmembrane helical bundle is the primary site for allosteric modulators. Another significant uniqueness is that these receptors are obligate dimers by virtue of a disulfide bond between their VFDs.

Experimental high-resolution structures exist for the 7TMD of mGluR_1_ (Wu et al., 2014) and the 7TMD and VFT of mGluR_5_ (Doré et al., 2014; Christopher et al., 2015, 2018; Koehl et al., 2019). There are not, however, published high-resolution structures of the 7TMD of mGluR_2_ and mGluR_3_, although their VFT domain has been determined by X-ray crystallography (Muto et al., 2007). Thus, we report here two recent MD-based studies, one for mGluR_1_ and another for mGluR_5_, both using their high-resolution experimental structures as starting conformations. To investigate mGluR_1_ allosteric modulation mechanism at an atomic level of detail appropriate for designing potent NAMs of this receptor, Bai and Yao carried out both biased and unbiased MD simulations on wild-type mGluR_1_ dimer, as well as its T815M and Y805A mutants, in complex with a NAM known as FITM (4-fluoro-*N*-(4-(6-(isopropylamino)pyrimidin-4-yl)thiazol-2-yl)-*N*-methylbenzamide), and embedded in a POPC lipid membrane environment (Bai and Yao, 2016). The simulations used the CHARMM27 force field for the protein and lipid, and the CGenFF force field for FITM. The unbinding PMF, calculated using an adaptive biasing force, revealed an intermediate ligand-binding state along the NAM-mGluR1 dissociation path, stabilized by interactions with residues S735, T748^ECL2^, C746^ECL2^, K811^7.28^ (renumbered K811^7.29^ according to Isberg et al., 2015) (Bai and Yao, 2016). While hydrogen bonding between FITM and Y805 was identified as a major contributor to stable ligand binding at the crystallographically determined allosteric site, ligand hydrogen bonding to T748 was found to be a crucial factor in stabilizing FITM in an intermediate binding site (Bai and Yao, 2016). Finally, weak interaction analysis of the stabilizing and destabilizing non-covalent interactions using unbiased MD simulations corroborated the importance of van der Waals and hydrogen bond interactions between Y805 and T815 residues in stabilizing the ligand at the binding site.

In another recent unbiased MD investigation, the structural basis for the binding of several NAMs in preclinical or clinical development (mavoglurant, dipraglurant, basimglurant, STX107, and fenobam), as well as three additional NAMs (MPEP, 51D, and 51E) at mGluR5, was elucidated (Fu et al., 2018). The simulations used an explicit POPC membrane in which to embed the ligand-mGluR5 complexes. The force field parameters of the protein atoms were based on the AMBER ff14SB force field, while the force field parameters of the lipid atoms used the Lipid14 force field and ligands were parametrized with the GAFF force field using the Antechamber program. To begin, five NAMs (dipraglurant, basimglurant, STX107, fenobam, and MPEP) were docked to the mGluR5 receptor allosteric site and short 100 ns MD simulations were used to assess the stabilities of the predicted docked poses. Using the final 50 ns of these trajectories, MM/GBSA calculations were carried out to rank the ligands according to their binding free energies. Using the per-residue free energies, eleven residues – I625^2.46^, I651^3.36^, S654^3.39^, P655^3.40^, L744^5.44^, W785^6.50^, F788^6.53^, M802^7.32^ (renumbered M802^7.33^ according to Isberg et al., 2015), V806^7.36^ (V806^7.37^ as per Isberg et al., 2015), S809^7.39^ (S809^7.40^ as per Isberg et al., 2015), and A810^7.40^ (A810^7.41^ as per Isberg et al., 2015) – were identified as the main contributors to the stable binding of all studied NAMs to mGluR_5_. The apolar nature of most of these eleven residues further suggested that ligands with hydrophobic scaffolds might be better mGluR5 binders.

## Concluding Remarks

In this work, we have reviewed recent MD-based investigations of a number of GPCRs that are currently in the spotlight for pain management or to treat or prevent OUD clinical manifestations. With the continued advancements in both computer hardware and MD simulation software, as well as sophisticated tools for analysis of increasingly larger datasets generated by MD simulations, atomic-level insights into the dynamical behavior of GPCRs involved in important pharmacological mechanisms are expected to contribute more and more to the rapid development of therapeutics in response to the opioid crisis.

## Author Contributions

JR and MF wrote the manuscript.

## Conflict of Interest Statement

The authors declare that the research was conducted in the absence of any commercial or financial relationships that could be construed as a potential conflict of interest.
